# A *Shigella flexneri* Virulence Plasmid Encoded Factor Controls Production of Outer Membrane Vesicles

**DOI:** 10.1534/g3.114.014381

**Published:** 2014-11-05

**Authors:** Saima Sidik, Haila Kottwitz, Jeremy Benjamin, Julie Ryu, Ameer Jarrar, Rafael Garduno, John R. Rohde

**Affiliations:** * Department of Microbiology and Immunology, Dalhousie University Halifax, NS, Canada B3H 4R2; † Canadian Food Inspection Agency, Dartmouth Laboratory, Dartmouth, NS Canada B3B 1Y9

**Keywords:** *Shigella*, outer membrane vesicles, virulence

## Abstract

*Shigella* spp. use a repertoire of virulence plasmid-encoded factors to cause shigellosis. These include components of a Type III Secretion Apparatus (T3SA) that is required for invasion of epithelial cells and many genes of unknown function. We constructed an array of 99 deletion mutants comprising all genes encoded by the virulence plasmid (excluding those known to be required for plasmid maintenance) of *Shigella flexneri*. We screened these mutants for their ability to bind the dye Congo red: an indicator of T3SA function. This screen focused our attention on an operon encoding genes that modify the cell envelope including *virK*, a gene of partially characterized function. We discovered that *virK* is required for controlled release of proteins to the culture supernatant. Mutations in *virK* result in a temperature-dependent overproduction of outer membrane vesicles (OMVs). The periplasmic chaperone/protease DegP, a known regulator of OMV production in *Escherichia coli* (encoded by a chromosomal gene), was found to similarly control OMV production in *S. flexneri*. Both *virK* and *degP* show genetic interactions with *mxiD*, a structural component of the T3SA. Our results are consistent with a model in which VirK and DegP relieve the periplasmic stress that accompanies assembly of the T3SA.

*Shigella* is a genus of pathogenic bacteria that causes the diarrheal disease shigellosis, which places an onerous and ongoing burden on the developing world. A study performed in 2006 that focused on Southeast Asia showed that the incidence of shigellosis in this part of the world has not subsided and that there is a high incidence of Shigellae that is multidrug resistant (von Seidlein *et al.* 2006). Hallmarks of shigellosis include high fever and a mucopurulent diarrhea with blood. The latter symptom is a result of bacterial invasion and destruction of colonic epithelium.

*Shigella flexneri* contains a 220-kb virulence plasmid encoding approximately 100 genes (Buchrieser *et al.* 2000). This plasmid encodes components of a Type III Secretion System and its effectors, which are necessary for invasion into epithelial cells (Sansonetti *et al.* 1982), survival inside macrophages, and a myriad of effects that modulate the host immune response (Parsot 2009; Phalipon and Sansonetti 2007). Many of the genes encoded by the virulence plasmid are uncharacterized or poorly characterized. The Type 3 Secretion Apparatus (T3SA) of *S. flexneri* is well-defined due partly to the fact that *Shigella* spp. bind the dye Congo red when the T3SA is active, providing a phenotype that is correlated with Type III secretion and virulence (Parsot *et al.* 1995; Sakai *et al.* 1986). The mechanism by which Congo red induces activity of the T3SA is unknown; it is presumed that proteins secreted from *S. flexneri* bind Congo red resulting in a red colony. The T3SA spans the bacterial cell envelope to create a structure capable of transporting effectors from inside the bacterium to the host cell cytosol. In Gram-negative bacteria, the cell envelope consists of the inner membrane, the cell wall, which is composed of a layer of heteropolymer glycan chains cross linked by amino acids (peptidoglycan), and the bacterial outer membrane, which is comprised of a phospholipid inner leaflet and a glycolipid outer leaflet (reviewed in (Silhavy *et al.* 2010)).

Gram-negative bacteria possess multiple envelope stress responses (ESRs) that maintain envelope integrity. These ESRs can be triggered by separate environmental insults and often are interconnected. Accumulation of unfolded proteins in the periplasm triggers activation of an alternate sigma factor, sigma E, that controls gene expression directed toward proper folding of proteins and repair of envelope damage. The production of outer membrane vesicles (OMVs) has been shown to represent a unique ESR (McBroom and Kuehn 2007). OMV production is increased in cells that cannot adequately deal with periplasmic stress, as in the case of deletions of the dual function DegP chaperone/protease (McBroom and Kuehn 2007). Hypervesiculating mutants show reduced fitness when combined with mutations that limit OMV production, indicating OMV production is required for maintaining envelope integrity (Schwechheimer and Kuehn 2013). OMVs can play a role in pathogenesis because they can transport toxins or deliver inflammatory components of the cell envelope to host cells (Kulp and Kuehn 2010).

Collections of deletion mutants, or “deletion collections,” are commonly used to identify novel gene functions by researchers who study organisms such as *Saccharomyces cerevisiae* (Giaever *et al.* 2002) or *Drosophila melanogaster* (Ryder *et al.* 2004). Deletion collections and similar tools created for the pathogens *Salmonella enterica* and *Staphylococcus aureus* have been used to discover new virulence genes and aid in development of antimicrobial agents (Donald *et al.* 2009; Santiviago *et al.* 2009). Here we report the creation of the pWR100 collection: a collection of precise mutants encompassing all genes encoded by the virulence plasmid of *Shigella flexneri*. Furthermore, we demonstrate the utility of the pWR100 collection by using it to identify new phenotypes associated with *virK*, a virulence plasmid-encoded gene. We find that loss of *virK* is associated with defects in T3SA function and hypervesiculation, suggesting that VirK supports T3SA function.

## Experimental procedures

### Bacterial strains and growth conditions

A streptomycin-resistant strain of *Shigella flexneri* serotype 5a (M90T-Sm) was used as the parent strain for all mutants in the pWR100 collection (Onodera *et al.* 2012). *S. flexneri* was routinely cultured in or on Trypticase soy broth (TSB) plus 0.01% Congo red, with or without 20 mg/mL agar. Tetracycline was used at a concentration of 5 μg/mL to select for the tetracycline resistance cassette (*tetRA*). Ampicillin, kanamycin, and gentamicin were used at 100 μg/mL, 25 μg/mL and 15 μg/mL, respectively, to maintain plasmid selection when applicable.

### Plasmid construction

The pWR100 collection was created using modified versions of the plasmids pKD46 (Datsenko and Wanner 2000) and pCP20 (Cherepanov and Wackernagel 1995). Two plasmids, pRR008 and pRR007, were created based on pKD46, which encodes the machinery necessary for λ red−mediated recombination. In both cases, pKD46 was combined with the plasmid pBBR1MCS-2 (Kovach *et al.* 1995) containing the gene *sacB* from pJQ200KS (Quandt and Hynes 1993) cloned into its *Bam*HI site. Both plasmids were linearized using *Spe*I, and the two fragments were ligated together. In the case of pRR008, the kanamycin resistance gene originally encoded by pBBR1MCS-2 was replaced by a gentamicin resistance gene from the plasmid pPH1J1 (Hirsch and Beringer 1984) by homologous recombination. The protein encoded by *sacB*, Levansucrase, can be used for counterselection against pRR007 and pRR008 when grown on sucrose. pRR007 and pRR008 are mobilizable in host strains that provide RK2 transfer functions *in trans*, due to mobilization machinery provided by the fragment isolated from pBBR1MCS-2.

pRR003 is a modified version of the plasmid pCP20, which encodes flippase machinery used to promote recombination between FRT sites. pRR003 was constructed using a 3.4-kb fragment excised from pCP20 using the enzymes *Bam*HI and *Xho*I. This fragment was ligated to a 5.3-kb fragment of the plasmid pBB1MCS-2 produced through cleavage with the same enzymes. The gene *sacB*, isolated from pJQ200KS using polymerase chain reaction (PCR), was cloned into the *Bam*HI site of pRR003 to facilitate counterselection. pRR003 is mobilizable in host strains that provide RK2 transfer functions *in trans*.

The plasmid *tetRA*-pGEM was created using the tetracycline resistance gene *tetRA* from the DNA fragment TH2788 (Karlinsey 2007). *TetRA* was first amplified by PCR using the primers Tet 2F-*Pst*I and Tet 2R-*Pst*I (Supporting Information, Table S1) and cloned into pGEM-T Easy using TA cloning. Using this plasmid as template, *tetRA* was again amplified using the primers T3 LR1R *Hin*dIII and T7 LR1F *Kpn*I (Table S1) and cloned into pGEM-T Easy using TA cloning, creating the plasmid “*tetRA*-pGEM.” The primers T3 LR1R *Hin*dIII and T7 LR1F *Kpn*I facilitated the addition of FRT sequences to *tetRA*, as well as sequences known as “P1” and “P2.” Descriptions of plasmids used for complementation are described in Table S2, and the relevant primer sequences can be found in Table S1.

### λ red−mediated recombination and *E. coli*–*S. flexneri* conjugation

Linear fragments of DNA used to replace genes on pWR100, or “knock-out cassettes,” were created by PCR using *tetRA*-pGEM as template and primers containing the P1 and P2 sequences (Table S1) as well as 50 bp of homology to the sequence flanking the region to be deleted. In the case of forward primers, this region of homology began 47 bp upstream of the start codon of the gene in question and ended directly after the start codon. In the case of reverse primers, this region started 29 bp downstream of the gene in question and ended 21 bp into the coding sequence. In this manner, the nucleotides encoding the methionine at position one, plus the last six amino acids and the stop codon, were maintained. Knock-out cassettes were purified using a QIAGEN PCR purification kit.

pRR008 was transformed into electrocompetent M90T-Sm, and the resulting strain was used as a parent strain for all deletion mutants. Selection for pRR008 was maintained using 15 μg/mL gentamicin. A two-milliliter culture of M90T-Sm pRR008 was grown overnight, then subcultured at a ratio of 1:100 in 50 mL of medium containing 1% L-arabinose the next morning. Subcultures were grown until the OD_600_ reached between 0.4 and 0.6, at which time the bacteria were pelleted and resuspended twice in 25 mL of ice-cold water. All centrifugation steps were performed using an Eppendorf 5810R centrifuge with an A-4-81 rotor at 3,220 g and 4° for 10 min, unless otherwise stated. The bacteria were then pelleted a fourth time and resuspended in approximately 500 μL of water. Then, 50 μL of the resuspended bacteria was added to a 2 mM electroporation cuvette along with 15 μL of purified knock-out cassette. The bacteria were electroporated, then allowed to recover for 2 hr in 1 mL of TSB at 37° with shaking at 200 rpm. The bacteria were then pelleted at 2300 g for 5 min at room temperature and plated on a TSB Congo red plate containing tetracycline and grown overnight at 37° to select for integration of the knock-out cassette. Loss of pRR008 was selected for by growth on TSB Congo red plates containing 5% sucrose.

Integration of the knock-out cassette at the desired location was confirmed by PCR using primers for the P1 or P2 sequence in the knock-out cassette with a primer upstream (in the case of P1) or downstream (in the case of P2) of the region being deleted. Obtaining a product from such a PCR indicated correct integration of the knock-out cassette.

Tetracycline resistance genes were removed by catalyzing recombination between the flanking FRT sites using the plasmid pRR003. This plasmid was introduced into mutants by electroporation, as described previously, or by mating using the *E. coli* strain S17-1λ pir. In the case of mating, *S. flexneri* was mixed with S17-1λ pir pRR003 in approximately a 1:1 ratio, spotted on TSB and allowed to incubate at 37° for 5 hr. The mix of bacteria was then plated on M9 minimal media plate supplemented with 10 μg/mL nicotinic acid plate containing kanamycin (to select for *S. flexneri* containing pRR003) and incubated at 37° overnight. Colonies were patched onto TSB Congo red plates containing kanamycin, with and without tetracycline. Strains that were susceptible to tetracycline were selected, as this indicated loss of the tetracycline resistance gene. In the case of electroporation, *S. flexneri* was allowed to recover for 90 min, then plated on TSB Congo red plates containing kanamycin. Single colonies were patched onto TSB Congo red plates containing kanamycin, with and without tetracycline.

### Construction of a nonpolar *ipaH9.8* mutant

The *ipaH9.8ΔNterm*::*tetRA* mutant was created as described above with the exception that only approximately the middle third of *ipaH9.8* (amino acids 183−337, inclusive) was replaced by the knock-out cassette. The primers used to create such a knock-out cassette are listed in Table S1 as *ipaH9.8*-Ntermdelfor and *ipaH9.8*-C337delrev.

### Congo red screens

Strains from the pWR100 collection were pinned onto 14-cm diameter, round TSB Congo red plates in triplicate. Strains were pinned in different locations on each of the three plates to avoid edge effects. As controls for the screen of the pWR100 collection, the same 96 strains were pinned onto Luria broth plates without Congo red, and 94 strains from the Keio *E. coli* deletion collection were pinned onto a Congo red TSB plate. Strains were grown for 16 hr at 37°. Plates were imaged using a VersaDoc imaging system from BioRad with an excitation wavelength of 430 nm and an emission wavelength of 503 nm. Densitometry was performed using Image Lab software from BioRad. The density of the spot corresponding to each strain was determined using local background subtraction, these values were averaged for each strain, and the average for each mutant was subtracted from the average for M90T-Sm to obtain a score in “CR units.”

### Reverse transcriptase quantitative PCR (RT-qPCR)

**Figure 1:**
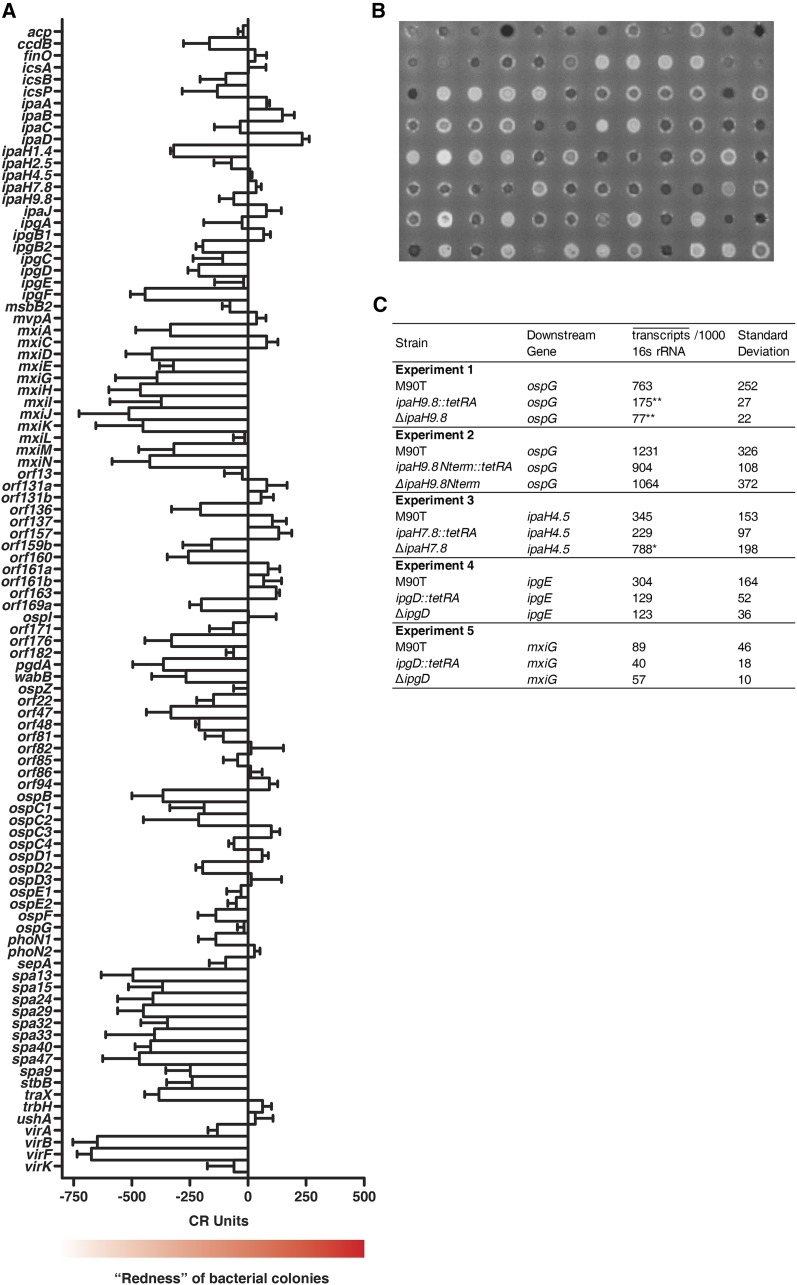
Fidelity of the deletion collection to established phenotypes. (A) Results from a screen for the ability of mutants from the pWR100 collection to bind the dye Congo red. CR units are defined as the density of wild-type *S. flexneri* (M90T-Sm) minus the density of the indicated mutant. Error bars indicate standard deviation of three replicates. (B) Ninety-six *S. flexneri* mutants were grown on Trypticase soy broth containing Congo red, then photographed using an excitation wavelength of 430 nm and an emission wavelength of 503 nm. (C) Reverse transcription quantitative polymerase chain reaction was performed to establish the effect of several mutations on transcription of downstream genes. Three replicates were used to establish the mean number of transcripts per 1000 16S rRNA transcripts and a standard deviation. Groups of strains that were analyzed together are separated by horizontal lines. Probability of being the same as M90T-Sm: *0.05 > *P* > 0.01, **0.01 > *P* > 0.001 (see the section *Materials and Methods* for statistical methods).

Bacterial cultures were grown overnight at 37° with shaking at 200 rpm, then diluted at a ratio of 1:100 in TSB and grown for an additional three hours under the same conditions. In experiments 1, 2, and 3 (Figure 1C) Congo red was added to the cultures to a final concentration of 0.05% after 2 hr, and the cultures were then grown for an additional hour. RNA was extracted using an RNeasy mini kit from QIAGEN (cat. no. 74014). cDNA was then synthesized from 1 μg of total cellular RNA using qScript cDNA SuperMix from Quanta Biosciences (cat. no. 95048-25). Two microliters of cDNA (equivalent to 100 ng of total RNA) was used as template for qPCR using PerFeCta SYBR Green FastMix from Quanta Biosciences (cat. no. 995072-250). Samples were run on a RotorGene 3000 from Corbett Research. Primer sequences are listed in Table S1 as the name of the gene they amplify, followed by “RT1,” followed by either “F” for “forward” or “R” for “reverse.”

### Gentamicin protection assays

HeLa cells were cooled to room temperature for 15 min before infection, then infected at an MOI of 10 using strains of *S. flexneri* containing the afimbrile adhesin E gene (*afaE*) from *E. coli* (Labigne-Roussel *et al.* 1984). Infected cells were allowed to incubate at room temperature for 15 min, then incubated at 37° with 5% CO_2_ for 1 hr. Medium was then removed from the cells and replaced with Dulbecco’s Modified Eagle’s medium containing 10% fetal bovine serum and 50 μg/mL gentamicin, and the cells were incubated at 37° with 5% CO_2_ for an additional hour. Cells were washed once with phosphate-buffered saline, then lysed in 500 μL of NP-40 lysis buffer (0.1% NP-40; 50 mM Tris-HCl, pH 7.5; 5 mM ethylenediaminetetraacetic acid; 10% glycerol, 100 mM sodium chloride). Fifty microliters of 1:10 serial dilutions of the cell lysates were plated on TSB agar media containing Congo red and incubated at 37° overnight. Colony-forming units were counted the next day.

### Secretion assays

Bacterial strains were grown to mid-log phase, and approximately 4 × 10^8^ bacteria were pelleted at 3220*g* for 10 min and resuspended in 1 mL of phosphate-buffered saline. Congo red was added to a final concentration of 425 μg/mL unless otherwise indicated, and the bacteria were incubated at 37° or 30° for 30 min. Bacteria were pelleted at 3000 g for 10 min, then the supernatant was collected and filtered through a 0.45-μM filter. Bacterial pellets were resuspended in 250 μL of sodium dodecyl sulfate (SDS) sample buffer (50 mM Tris-HCl, pH 6.8; 2% SDS; 100 mM dithiothreitol; 10% glycerol) and boiled for 5 min. Supernatant samples were mixed with SDS sample buffer in a 3:1 ratio of sample to buffer and boiled for 5 min before gel loading. All samples were run on 10% SDS-polyacrylamide gel electrophoresis gels. Gels were stained using either a silver stain kit from BioRad (cat. no. 161-0449), in the case of supernatant samples, or Coomassie in the case of bacterial pellet samples.

### β-galactosidase assays

Bacterial strains were grown to mid-log phase, then 500 μL of culture was transferred to a 1.5-mL tube and incubated at 37° for 30 min. One hundred microliters of bacterial culture was then mixed with 500 μL of Z-buffer (0.06 M Na_2_HPO_4_⋅7H_2_O, 0.04 M NaH_2_PO_4_⋅H_2_O, 0.01 M KCl, 1 mM MgSO_4_⋅7H_2_O, 0.05 M β-mercaptoethanol), 10 μL of 0.1% SDS, and 10 μL of chloroform. Cells were aspirated, then incubated at room temperature for 15 min. One hundred microliters of bacterial solution was mixed with 25 μL of ONPG (10 mg/mL), and the cells were incubated at room temperature until the mixture produced a yellow color. The reaction was stopped through the addition of 50 μL of 1 M Na_2_CO_3_, and the OD_420_ was read using an Eon microplate spectrophotometer from BioTek. The OD_600_ of each bacterial strain was also obtained to allow normalization for the number of cells.

### Determination of periplasm protein content

Periplasm protein content was determined using a Bradford assay as described (Schwechheimer and Kuehn 2013).

### Electron microscopy

Unfixed whole cells, taken directly from broth cultures with minimum disturbance, were allowed to interact with the surface of Formvar and carbon-coated copper grids for up to 5 min. After excess fluid was drawn from the surface of grids with a triangular piece of filter paper, grids were floated on drops of a saturated and unbuffered solution of ammonium molybdate for 20−30 sec. Grids were then air dried and immediately observed in a JOEL JEM-1230 transmission electron microscope equipped with a Hamamatsu ORCA-HR high-resolution (2K by 2K) digital camera. Images captured were saved as TIFF files. For quantification of OMVs, images from the *virK* or *degP* mutants or wild type bacteria grown at 37° were coded and analyzed by counting the proportion of bacteria that were associated with OMVs and expressed as a percentage.

### Statistics

In Figure 1C, three biological replicates were performed for each experiment. One-way analyses of variance (ANOVAs) were performed for each experiment, and, if significance was concluded, these were followed by Dunnett’s tests. *P*-values resulting from one-way ANOVAs were as follows: Expt. 1: 0.0025, Expt. 2: 0.4423, Expt. 3: 0.0103, Expt. 4: 0.1210, Expt. 5: 0.1969.

**Figure 2:**
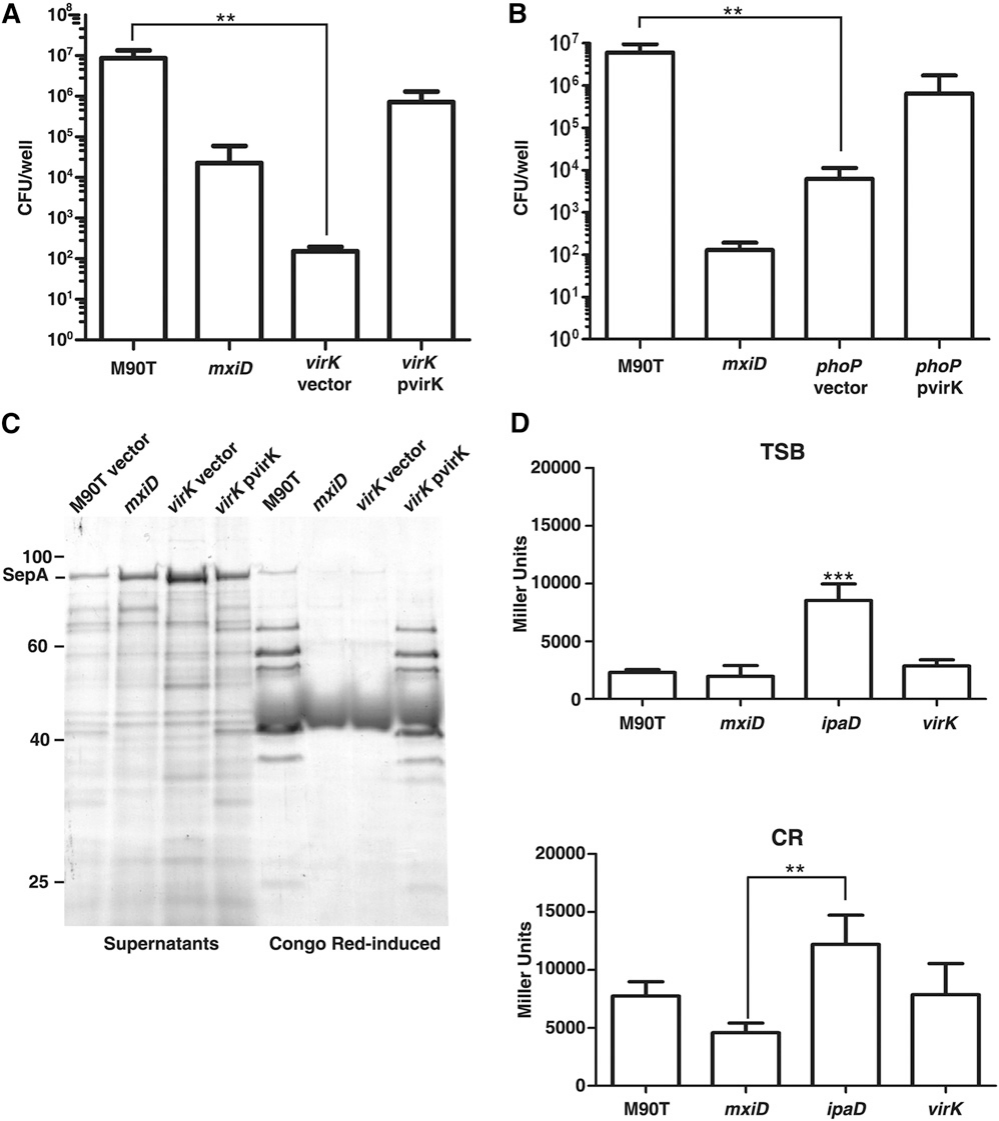
A *virK* mutant is deficient for entry of epithelial cells and displays an aberrant protein secretion profile. Gentamicin protection assays were performed using HeLa cells, and the number of colony-forming units (CFU) recovered from each well of infected HeLa cells was determined. (A) Invasion of a *virK* mutant harboring the plasmid pBluescript was compared with that of wild-type *S. flexneri*, a *mxiD* mutant, and a *virK* mutant containing the plasmid pvirK which encodes *virK*. A one-way analysis of variance (ANOVA) was performed followed by a Dunnett’s test. (B) Invasion of a *phoP* mutant was compared with that of wild-type *S. flexneri* using a Mann-Whitney test. **0.001 < *P* < 0.01. (C) Coomassie-stained sodium dodecyl sulfate polyacrylamide gel electrophoresis (SDS-PAGE) gels show the profiles of secreted proteins from wild-type *S. flexneri* bearing an empty vector control (pBluescript) along with the mutants *mxiD*, *virK* bearing an empty vector control (pBluescript) and *virK* bearing pvirK. Coomassie-stained SDS-PAGE gels on which crude extracts from bacteria used to collect secreted proteins have been run serve as loading controls (Figure S3). (D) Results from β-galactosidase assays in which the indicated bacterial strains contain a plasmid encoding *lacZ* under the control of a Type 3 Secretion Apparatus−responsive promoter. Error bars indicate standard deviation of five replicates. ***0.001 > *P* > 0.0001, 0.001 , P , 0.01 as assessed using a one-way ANOVA.

In Figure 2, A and B, three biological replicates were performed for each experiment. Invasion of a *virK* mutant was compared with that of the wild type using a one-way ANOVA followed by a Dunnett’s Multiple Comparison Test. Invasion of a *phoP* mutant was compared with that of wild-type using a one-way ANOVA followed by a Dunnett’s multiple comparison test, **P ≤ 0.0094. In Figure 2D, The optical densities (ODs) of five biological replicates were measured at 420 nm and 600 nm. The OD_600_ of a “blank” well containing only media was subtracted from that of each experimental well. A strain of wild-type *S. flexneri* containing a plasmid encoding *lacZ* with no promoter also was included in the experiment. The average OD_420_ of this strain was subtracted from all other strains. Miller units were calculated using the equation:

$$\mathrm{Miller}\;\mathrm{Units}=1000\times OD_{420}/\left(OD_{600}\mathrm{\times volume}\left(\mathrm{mL}\right)\mathrm{\times development}\;\mathrm{time}\left(\min\right)\right)$$

A one-way ANOVA was performed followed by a Dunnett’s test. The *P*-value resulting from the one-way ANOVA was < 0.0001. In Figure 4, 10 biological replicates were performed. One-way ANOVA followed by a Bonferroni’s multiple comparison test was used, ***P ≤ 0.001. In Figure 5, C and D, three biological replicates were used in each experiment. One-way ANOVA followed by a Dunnett’s multiple comparison test was used. In Figure 5C, protein concentration was compared with *virK*. In Figure 5D, protein concentration was compared with *degP mxiJ*.

## Results

### Construction of the pWR100 collection

We used λ red−mediated recombination (Figure S1A) (Datsenko and Wanner 2000) to create an ordered array containing mutants for 99 of the 106 genes encoded by pWR100, as identified according to Genbank accession number AL391753 (Buchrieser *et al.* 2000). Seven mutants essential for maintenance of the virulence plasmid are not included in the pWR100 collection, namely *ccdA*, *mvpA mvpT*, *repB*, *copA*, *tapA*, and *repA* (Bahassi *et al.* 1999; Blomberg *et al.* 1992; Cevallos *et al.* 2008; Sayeed *et al.* 2000). Figure S1B, adapted from Buchrieser *et al.* (2000), displays the genes targeted for deletion in the pWR100 collection.

Each gene included in the pWR100 collection was replaced with a tetracycline resistance gene *tetRA* (Karlinsey 2007). A similar strategy was used previously by Baba *et al.* 2006 in the creation of the Keio *E. coli* deletion collection and has been established as an efficient method for creating a saturated collection of nonessential mutants. The *tetRA* cassette can easily be removed from pWR100 by using a plasmid-encoded *flippase* gene to catalyze homologous recombination between flanking FRT sites (Cherepanov and Wackernagel 1995). After removal of *tetRA*, a “scar sequence” encoding the amino acid sequence MIPGIRRPAVRSSTSLGSIGTSKQLQPT plus the last six amino acids of the gene targeted for replacement is encoded in place of the original gene.

### Analysis of the pWR100 deletion collection for Congo red binding

*Shigella* spp. bind the dye Congo red, and binding is associated with virulence and secretion of T3SA effectors (Benjelloun-Touimi *et al.* 1995). The ability to bind Congo red routinely is used to ensure that strains retain T3SA function after genetic manipulation, and has been used as an assay to investigate the structure of the T3SA (Kenjale *et al.* 2005). We measured the ability of the mutants of the pWR100 collection to bind Congo red (Figure 1A). We made the fortuitous discovery that the dye Congo red can be imaged at a wavelength of 503 nm when illuminated with a wavelength of 430 nm, providing a quantitative assay for this phenotype that is associated with T3SA function. This allowed us to obtain grayscale images of the mutant strains in which the color density of the colonies indicated the ability of the mutants to bind Congo red (Figure 1B). The mutant strains also were grown on media without Congo red to ensure that this effect was dependent on the presence of dye (data not shown). The ratio of the standard deviation to the average of the Congo red scores obtained from this control was less than that obtained from the *S. flexneri* deletion collection plated on Congo red-containing medium (about 0.12, as opposed to 0.2), indicating that Congo red is necessary to observe variation in the fluorescence of the mutants. *S. flexneri* mutants for whom Congo red phenotypes have been reported behaved as expected in a Congo red binding assay, indicating that our deletion strategy produces the expected results in terms of effector production and secretion. For example, an *ipaD* mutant, in which effector secretion is constitutive (Menard *et al.* 1994), was the most Congo red−positive strain screened. A mutant for *ospD1*, an antiactivator for the transcription factor MxiE (Parsot *et al.* 2005), also was more Congo red−positive than M90T-Sm. Mutants for the transcription factor-encoding genes *virB* and *virF* were among the most Congo red−negative strains (Adler *et al.* 1989). Many mutants for genes of unknown function were much more Congo red negative than the wild-type parent (M90T-Sm) (*e.g.*, *orf185* and *orf47*) and some mutants for genes of unknown function were more Congo red−positive than M90T-Sm (*e.g.*, *orf131a*, *orf131b*, and *orf163*). These genes may play roles in regulating effector secretion or may affect Congo red binding through a mechanism independent of T3SA function.

Our mutagenesis strategy was designed to minimize polar effects on downstream genes by maintaining an open reading frame in place of each deleted gene, both before and after removal of *tetRA*. To examine the efficacy of this system, we performed RT-qPCR on four genes using RNA isolated from mutants for genes upstream of these four genes (Figure 1C). Both mutants containing *tetRA* and those in which *tetRA* had been removed were analyzed. Of these four genes, one mutant, *ipaH9.8*, reduced transcription of the downstream gene, *ospG*, to a statistically significant degree. This effect was observed both with and without *tetRA*. Transcription of *ipaH4.5* was increased upon deletion of the upstream gene, *ipaH7.8*, although this effect was only observed after removal of *tetRA*. It is possible that moving the binding site for MxiE, the transcription factor that activates transcription of the *ipaHs*, closer to the *ipaH4.5* transcription start site causes a greater number of *ipaH4.5* messages to be successfully completed.

The reason for the polar effect of the *ipaH9.8* mutation on *ospG* is unknown. A smaller mutation that replaces approximately the middle third of *ipaH9.8* with the scar sequence left behind after removal of *tetRA* is not polar (Figure 1C Experiment 2). These results reflect mechanisms of *S. flexneri* gene regulation that are yet to be elucidated (Bongrand *et al.* 2012). The nonpolar *ipaH9.8* mutant is included in the pWR100 collection, with and without *tetRA*, under the name “*ipaH9.8ΔNterm*.” IpaH9.8 is an E3 ubiquitin ligase, and the catalytic ability of this Type III Secretion System effector depends on a cysteine residue found at amino acid position 337 (Rohde *et al.* 2007). This cysteine is included in the region that has been deleted, and so the ability of *ipaH9.8* to encode an active E3 ubiquitin ligase is eliminated in the *ipaH9.8ΔNterm* strain.

### Altered release of proteins to the culture supernatant in a *virK* mutant

Several mutants for undercharacterized genes were found to be deficient in their ability to bind Congo red (Figure 1A). One of these, *ipgF*, is encoded within a region of the virulence plasmid that encodes structural elements of the T3SA needle apparatus (Buchrieser *et al.* 2000). Removal of the *tetRA* cassette restored a Congo red positive phenotype to the *ipgF* mutant, suggesting that the *ipgF*::*tetRA* mutation had a polar effect that was alleviated when the *tetRA* cassette was removed. Removal of the *tetRA* cassette did not restore a Congo red phenotype to other mutants, including two mutants, *pgdA* and *virK*, that are in an operon that includes: *pgdA-wabB-virK-msbB2* (Buchrieser *et al.* 2000; Goldman *et al.* 2008; Kaniuk *et al.* 2004). We found that the *pgdA*::*tetRA* mutation resulted in a polar effect on the operon and that, in contrast to the *ipgF* mutant, the polar effect was still observed when the *tetRA* cassette was removed. We created a *pgdA*::*kan* mutant using the same oligos used to amplify the *tetRA* cassette. The *pgdA*::*kan* mutant did not show a polar effect on downstream genes (Figure S2). These results show that the construction of a complete and non-polar collection of mutants will require a systematic and comprehensive analysis of each gene.

The *virK* mutant was chosen for further analysis. We found that the *virK* mutant that we constructed was impaired for the ability to enter cultured epithelial cells and that *virK* expressed from a plasmid could restore invasion to near wild-type levels (Figure 2A). Transcription of the *pgdA-wabB-virK-msbB2* operon is largely controlled by the PhoP-PhoQ two-component system (Goldman *et al.* 2008). The PhoPQ system is often co-opted to control virulence programs and the PhoPQ regulon includes many factors that modify components of the cell envelope (Alteri *et al.* 2011; Prost and Miller 2008). PhoPQ is essential for virulence in *S. flexneri*, although its role in invasion of epithelial cells is ambiguous (Cai *et al.* 2011; Moss *et al.* 2000). We found that a *phoP* mutant was significantly impaired in its ability to invade epithelial cells in the conditions of our experiment (Figure 2B). We tested whether expression of *virK* from a constitutive promoter (*lac*) could restore the *phoP* invasion defect. The ability of a *phoP* mutant bearing a *virK* expressing plasmid was not increased to a statistically significant degree. We conclude that the invasion defect observed in a *phoP* mutant may be due to more than one gene under the control of PhoP.

The profile for proteins released to the culture supernatant in the *virK* mutant was altered compared to the parent strain (Figure 2C). The proteins secreted into the culture supernatant without addition of Congo red included different species than those secreted by wild-type *S. flexneri*. Also, in culture supernatants from *virK* we reliably see an increase in a species that corresponds to the autotransporter toxin SepA (Figure 2C and Figure S4) (Benjelloun-Touimi *et al.* 1995). We observed that the culture supernatant from the *virK* mutant induced with Congo red contained much fewer proteins than wild type and more closely resembled the culture supernatant from an *mxiD* mutant that does not assemble a T3SA. When a plasmid that constitutively expressed *virK* was introduced into the *virK* mutant, the secretion profile resembled the wild type. Mutations in a regulator of T3SA activity, *ipaD*, result in a similar phenotype: increased secretion of proteins to the culture supernatant without Congo red induction and an inability to invade epithelial cells (Menard *et al.* 1994).

To test the effect of *virK* on T3SA function, we examined the activity of a reporter gene that is controlled by the activity of the T3SA. We used a *lacZ* reporter gene under the control of the *ipaH9.8* promoter, which is known to be constitutive in an *ipaD* mutant (Le Gall *et al.* 2005). We found that transcription of the reporter gene was significantly increased in a *ipaD* mutant but not in a *virK* mutant (Figure 2D). These data indicate that mutation of *virK* does not trigger production of effectors that are under the control of the activity of T3SA and argue against VirK regulating the activity of T3SA.

We considered the possibility that the proteins in the culture supernatant of *virK* mutant cells may be due to lysis of a proportion of cells in the culture. We observed that the *virK* mutant had a growth rate that was the same as the parent strain. These data suggest that the release of proteins to the culture supernatant by the *virK* mutant was not due to deregulated T3SA or due to massive cell lysis.

### Release of proteins to the culture supernatant in a *virK* mutant is temperature dependent

**Figure 3:**
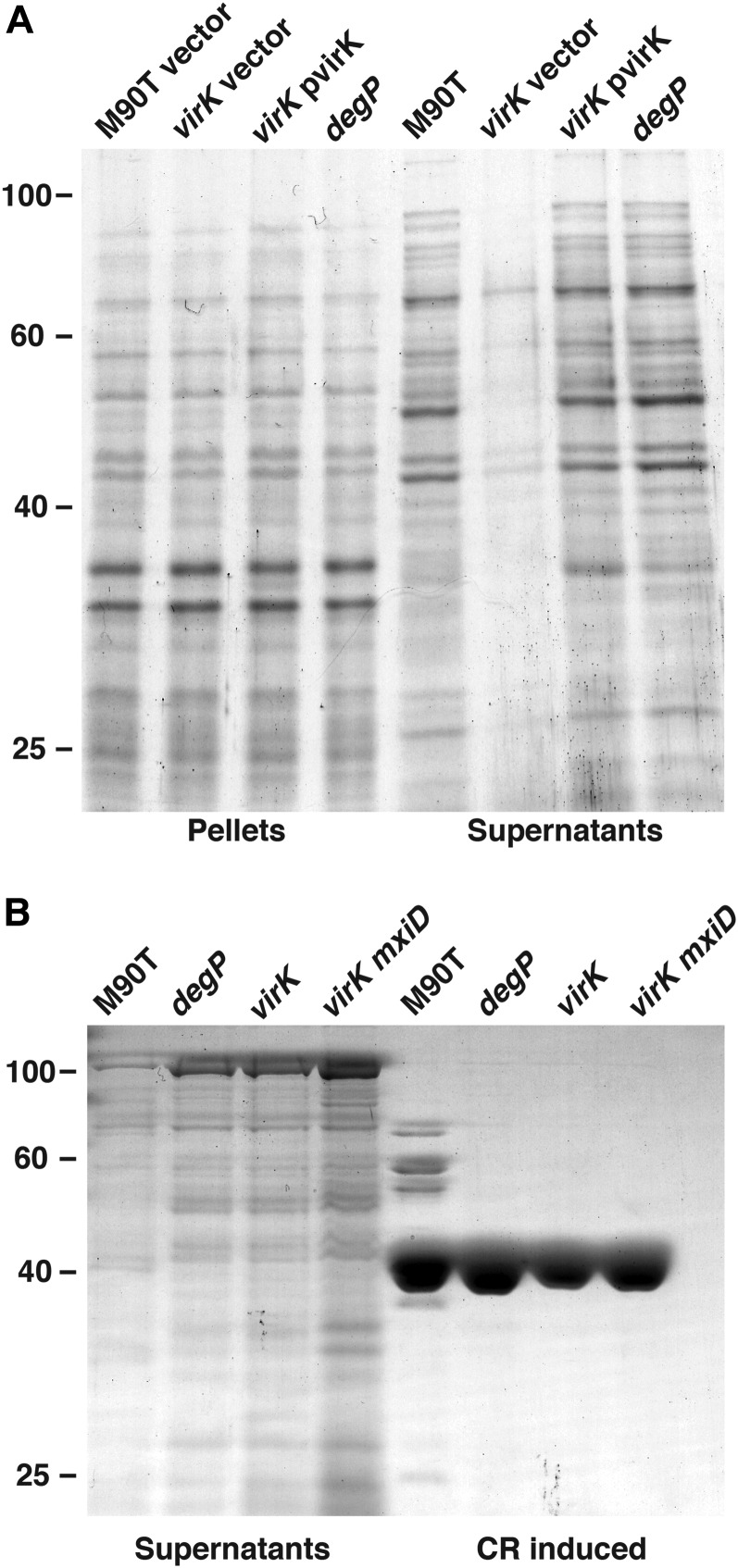
Aberrant secretion profile of *virK* mutant is temperature dependent. (A) Coomassie-stained sodium dodecyl sulfate polyacrylamide gel electrophoresis gels show the profiles of secreted proteins from wild-type *S. flexneri* along with the mutant *virK* or a mutant *degP*. Cultures were grown at 30°, and a sample was taken for preparation of a total protein (pellet). The cells were concentrated and proteins in the cell free supernatant were isolated using TCA precipitation. (B) Profile of secreted proteins from wild-type *S. flexneri* along with the mutants *virK*, *degP*, and *virK mxiD*. Cultures were grown at 37° and concentrated by centrifugation. Protein was collected from the cell free supernatant by TCA precipitation (Supernatants), the cells were resuspended in phosphate buffered saline and induced with Congo red, secreted proteins were collected (CR induced).

VirK is a periplasmic chaperone protein that preferentially interacts with misfolded proteins (Tapia-Pastrana *et al.* 2012). We hypothesized that the release of proteins to the culture supernatant observed in the *virK* mutant may be associated with assembly of the T3SA. Assembly of the T3SA is temperature-dependent, and does not occur at 30° (Adler *et al.* 1989). We performed secretion assays using *virK* and wild-type *S. flexneri* at 30° and 37°. We found that a *virK* mutant did not secrete proteins at 30°, providing a correlation between the *virK* mutant’s prolific secretion and the T3SA (Figure 3A). We observed a basal level of secretion of proteins to the supernatant in wild-type cultures in accord with previous findings (Blocker *et al.* 1999). By contrast, these proteins were absent in samples prepared from *virK* cells at 30° (Figure 3A).

DegP is a chaperone/protease that functions in the periplasm to aid in protein folding and to destroy ones that are improperly folded. Mutations in *S. flexneri degP* result in a phenotype similar to those that we had observed with *virK* (Nakata *et al.* 1992; Purdy *et al.* 2002). Both *virk* and *degP* were identified as virulence factors for *S. flexneri* based on their impaired intercellular spread, a process that requires both actin polymerization and T3SA (Bernardini *et al.* 1989; Page *et al.* 1999). We created a *degP* mutant and observed a release of proteins at 37° to the culture supernatant similar to that of *virK* (Figure 3B).

**Figure 4:**
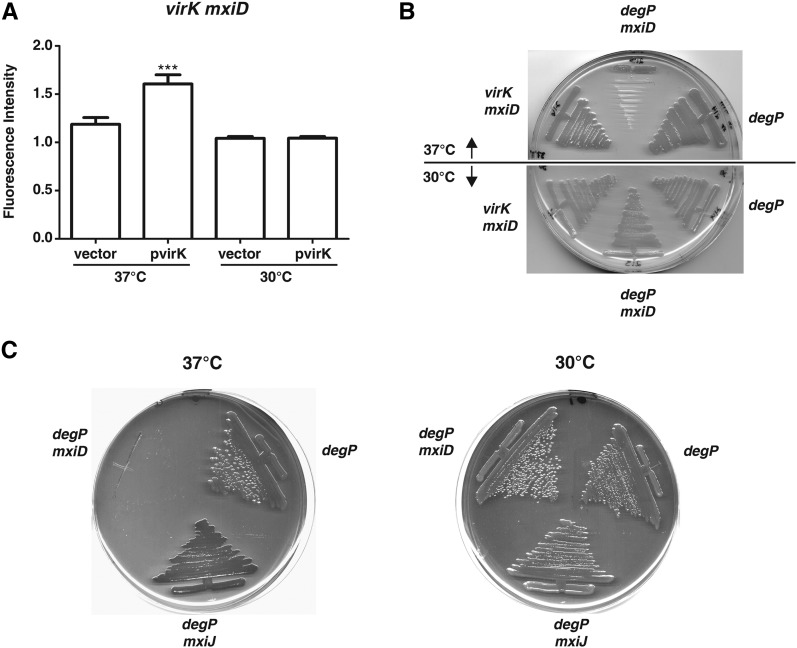
Mutations in *virK* or *degP* show synthetic phenotypes with structural components of the Type 3 Secretion Apparatus. (A) Congo red associated fluorescence of *virK mxiD* mutant strains bearing either an empty vector or one expressing *virK* were grown on Trypticase soy agar (TSA) containing Congo red at the indicated temperatures. The plates were photographed and analyzed according to Materials and Methods. (B). The indicated strains were streaked on TSA media and grown at the temperatures indicated. (C) The indicated mutants were grown on TSA containing Congo red at the indicated temperatures.

Because growth at temperatures where no T3SA is assembled suppressed the *virK* phenotype, we reasoned that deletion of genes required for T3SA assembly may similarly suppress release of proteins to the culture supernatant in *virK* cultures. We constructed a mutant bearing deletions in both *virK* and the secretin *mxiD* that encodes a component of the outer membrane ring of the T3SA and is required for T3SA assembly (Allaoui *et al.* 1993). Deletion of *mxiD* did not suppress the release of proteins to the culture supernatant in *virK* cultures (Figure 3B). Curiously, deletion of *virK* suppressed the Congo red−negative phenotype of the *mxiD* mutation. Expression of *virK* from a plasmid in *virK mxiD* mutants restored a Congo red negative phenotype (Figure 4A).

We examined possible genetic interactions between *degP* and *mxiD*. We found that *degP mxiD* mutants are viable at 30° but are unable to grow at 37° (Figure 4B). We tested genetic interactions between *degP* and another gene encoding a structural component of the T3SA, the base component *mxiJ*. We observe that a *degP mxiJ* mutant is viable at 30° and 37°. We observed that *degP mxiJ* double mutants were viable at 37° and that they were Congo red−positive (Figure 4C). We conclude that both *virK* and *degP* show genetic interactions with *mxiD* which encodes a component of the T3SA.

### VirK and DegP control the production of OMVs in *S. flexneri*

**Figure 5:**
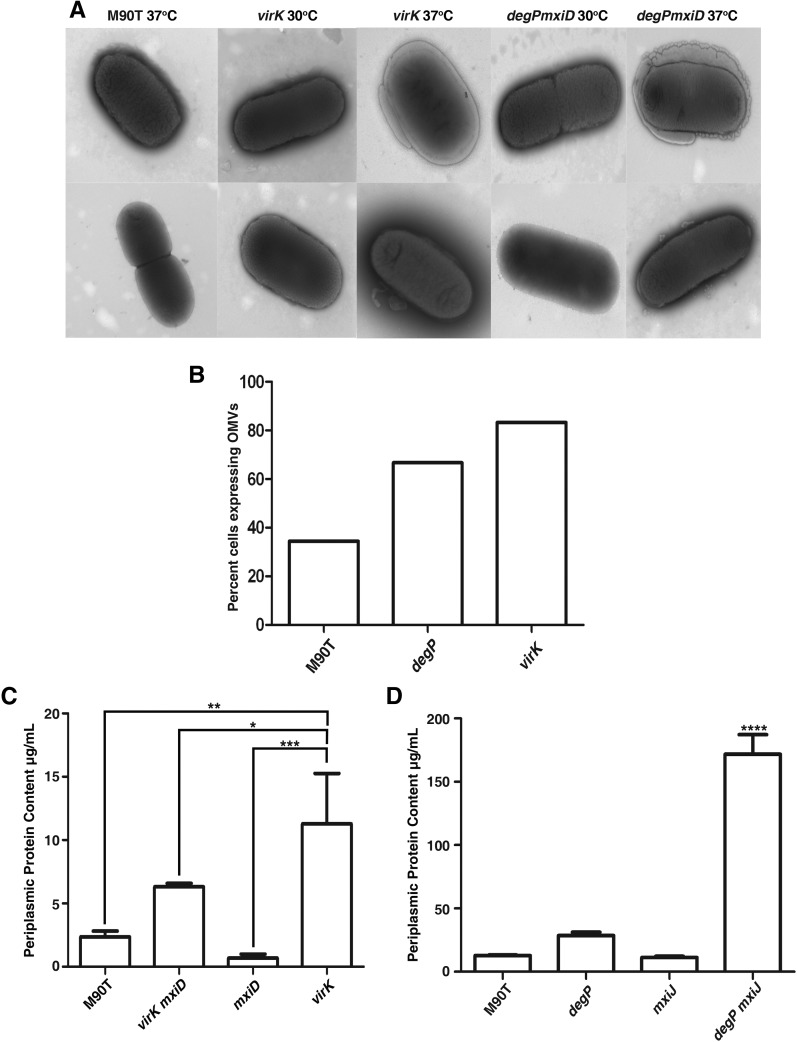
*virK* and *degP* mutants produce outer membrane vesicles (OMVs) in a temperature-dependent manner. (A) Wild-type and *virK* mutant *Shigella* were grown for 4 hr at 30° or 37° with selection from overnight cultures then negatively stained with ammonium molybdate and examined with TEM. *degP mxiD* was grown overnight at 30° then shifted to 37° and grown for 4 hr with selection before negative staining and examination with TEM. (B) For quantification of OMVs, images from the *virK* or *degP* mutants or wild-type bacteria grown at 37° were coded and analyzed by counting the proportion of bacteria that were associated with OMVs and expressed as a percentage. (C and D) The indicated strains were grown for 16 hr in LB at 37°, and the concentrations of periplasmic protein was determined using a Bradford assay (Schwechheimer and Kuehn 2013). ***0.0001 < *P* < 0.001, **0.001 < *P* < 0.01, *0.01 < *P* < 0.05 as assessed using a one-way analysis of variance.

In *E. coli*, mutation of *degP* results in an increased production of OMVs (McBroom and Kuehn 2007). OMV production is one type of ESR; vesicle production would be one way to explain the release of proteins to the culture supernatant in *virK* and *degP* mutants. We examined the idea that *virK* may influence production of OMVs. We used transmission electron microscopy of negatively stained, unfixed *S. flexneri* whole cells to observe production of OMVs. We observed very few vesicles in wild-type cells grown at 37° (Figure 5A). In contrast, many vesicles were associated with *degP* and *virK* cells grown at 37°, most cells were associated with multiple vesicles. No vesicles were observed in any strains in cells grown at 30°. We examined *degP mxiD* cells that had been grown at 30° and shifted to 37° for 4 hr. These cells produced many OMVs, and some cells were completely surrounded by vesicular structures (Figure 5A). We also counted the amounts of vesicles present in several fields around wild-type bacteria, *virK* mutants, and *degP* mutants. Blind scoring of the images from bacteria grown at 37° showed that a greater proportion of *virK* or *degP* mutants were associated with vesicles than the wild-type control (Figure 5B). The production of OMVs has been shown to be a novel envelope stress pathway that relieves stress that occurs in the periplasm as unfolded proteins accumulate. We examined the amount of proteins present in the periplasm in the various mutants. Mutation of *virK* resulted in a large increase in the amount of periplasmic proteins when compared with the wild type (Figure 5C). This increase could be suppressed by mutation of *mxiD*. We also observed an increase in periplasmic proteins in the *degP* and *degP mxiJ* mutant strains (Figure 5D). Taken together, these data indicate that *degP* and *virK* relieve envelope stress that accompanies growth at 37°.

## Discussion

The mutants that we constructed are easily cured of their *tetRA* cassette to produce markerless mutants. As all of these mutants were constructed in the same manner, in the same genetic background, this collection should prove useful for the construction of multiple gene deletions for the study of genetic redundancy, and for the rational development of vaccine candidates. Importantly, this collection is streptomycin resistant and is compatible with the *S. flexneri* oral model of infection in mice that has been developed by Bernardini and coworkers (Martino *et al.* 2005). Our strategy was designed to minimize polar effects; however, for reasons that we do not understand, some of the mutants do show polar effects on the expression of downstream genes. DNA topology in regions downstream of the transcription start site has been shown to exert effects on gene expression (Owen-Hughes *et al.* 1992). Also, multiple genes on pWR100 are produced by a phenomenon known as transcriptional slippage (Penno *et al.* 2006). A careful analysis of each mutant will be needed to achieve a comprehensive collection of nonpolar mutants. The construction of such a collection is an achievable goal and will benefit from open collaboration and communication in the *Shigella* research community.

We used an ordered array of mutants to screen for an *in vitro* phenotype associated with *S. flexneri* virulence *in vitro* and identified an operon with genes under the control of the PhoP-PhoQ two-component system (Goldman *et al.* 2008). The Congo red phenotype provides an *in vitro* assay associated with the activity of T3SA. Our results demonstrate that the Congo red phenotype is also associated with production of OMVs. We do not fully understand the role of OMVs in *Shigella* pathogenesis, but OMV production has practical applications for the production of vaccines or studies of innate immunity (Berlanda Scorza *et al.* 2012; Irving *et al.* 2014).

We provide evidence that *virK* and *degP* function to reduce the stress imposed by unfolded proteins in the periplasm. The T3SA is a macromolecular machine that spans both the inner and outer membranes, the controlled assembly of the T3SA requires remodeling of the cell envelope that invokes an ESR. This idea is clearly established in *Yersinia* , where T3SA assembly and the phage shock response are coupled (reviewed in (Flores-Kim and Darwin 2012). In this case, phage shock proteins B and C are essential during conditions in which a T3SA is assembled (Horstman and Darwin 2012). By analogy, deletion of *degP* or *virK* result in an increase in T3SA components and invoke an ESR that results in the production of OMVs. Both DegP and VirK have been implicated in *S. flexneri* virulence based on their requirement for cell-to-cell spread. Intracellular spread requires proper function of IcsA and mutations of either *degP* or *virK* result in impaired processing of IcsA (Purdy *et al.* 2007; Wing *et al.* 2005). Intracellular spread also requires T3SA activity (Page *et al.* 1999). The genetic interactions between *degP/virK* and components of the T3SA (*mxiD* and *mxiJ*) imply that DegP and VirK may contribute to cell-to-cell spread by influencing T3SA in addition to IcsA. The release of proteins to the culture supernatant in virK and *degP* mutants is most likely T3SA independent. The proteins are released independent of Congo red induction and the release of proteins is not accompanied by an increased expression of genes that are controlled by the activity of the T3SA. VirK was recently found to be a chaperone for the plasmid-encoded toxin (Pet) of Enteroaggregative *E. coli* that is secreted via an autotransporter mechanism (Tapia-Pastrana *et al.* 2012). Taken together, our data suggest that VirK serves a general quality control function for multiple weapons, including IcsA processing, toxin export, and T3SA assembly.

## Supplementary Material

Supporting Information
