# Supplementary material for: A Shigella flexneri Virulence Plasmid Encoded Factor Controls Production of Outer Membrane Vesicles
Source: G3 (Bethesda). 2014 Nov 5;4(12):2493–503. doi: 10.1534/g3.114.014381 (PMC4267944; doi:10.1534/g3.114.014381)
Supplement: Supporting Information [file supp_4_12_2493__index.html]

A Shigella flexneri Virulence Plasmid Encoded Factor Controls Production of Outer Membrane Vesicles — Supporting Information 

# A *Shigella flexneri* Virulence Plasmid Encoded Factor Controls Production of Outer Membrane Vesicles

## Supporting Information for Sidik *et al.*, 2014

**Files in this Data Supplement:**

- Figure S1 - .ai, 1 MB
- Figure S2 - Polarity of pgdA mutants. (.ai, 1017 KB)
- Figure S3 - Loading control of total protein extracts taken from cultures used for protein secretion profiles (Figure 2). (.ai, 1 MB)
- Figure S4 - Identification of SepA as major secreted species. (.ai, 1 MB)
- Table S1 - .ai, 993 KB
- Table S2 - .ai, 1 MB
