# Supplementary figures and images for: A Shigella flexneri Virulence Plasmid Encoded Factor Controls Production of Outer Membrane Vesicles
Source: G3 (Bethesda). 2014 Nov 5;4(12):2493–503. doi: 10.1534/g3.114.014381 (PMC4267944; doi:10.1534/g3.114.014381)

S1

A

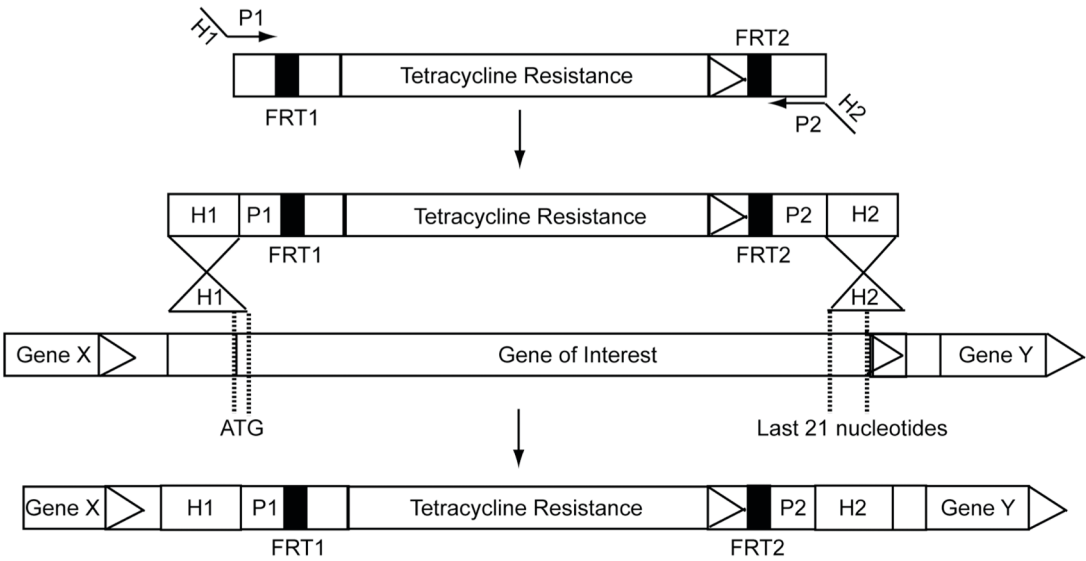

B

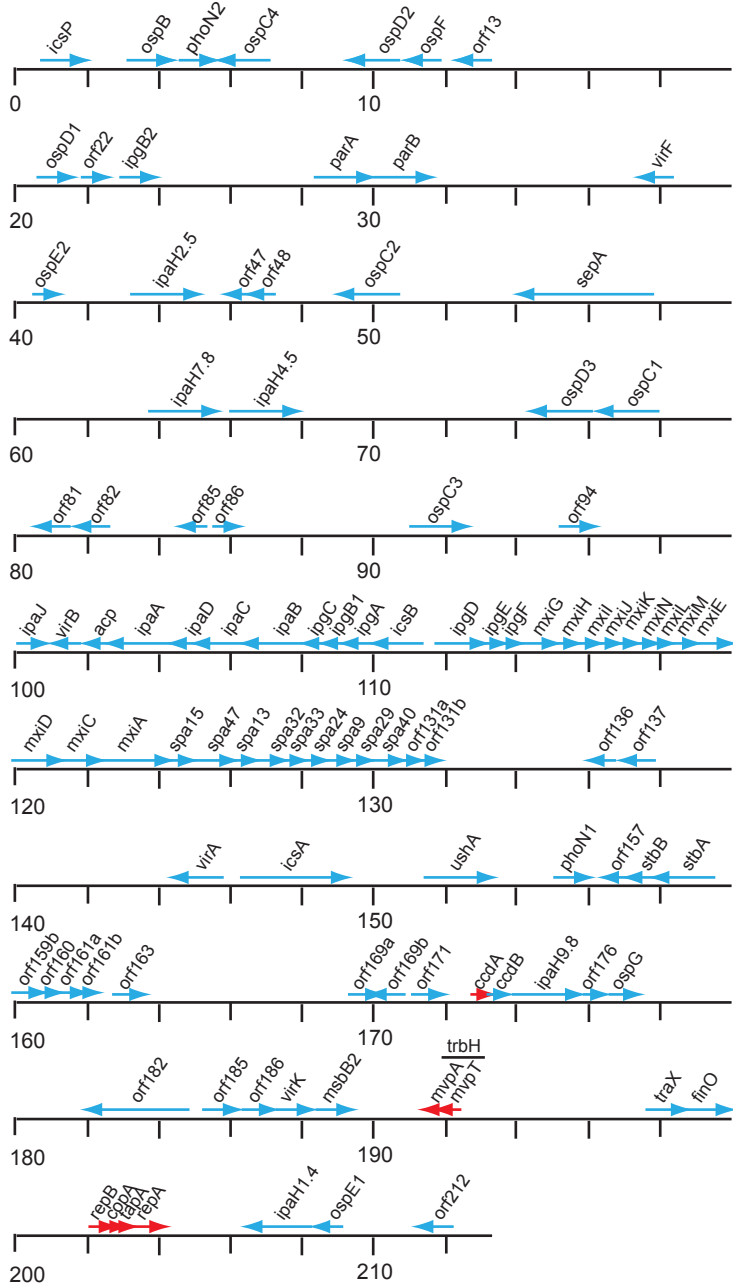

Supplement: Supporting Information [file supp_g3.114.014381_FigureS1.ps]
