# Supplementary material for: A Shigella flexneri Virulence Plasmid Encoded Factor Controls Production of Outer Membrane Vesicles
Source: G3 (Bethesda). 2014 Nov 5;4(12):2493–503. doi: 10.1534/g3.114.014381 (PMC4267944; doi:10.1534/g3.114.014381)
Supplement: Supporting Information [file supp_g3.114.014381_FigureS2.ps]

S2

Strain	Downstream Gene	transcripts /1000 16s rRNA	Standard Deviation
**Experiment 1**			
M90T	*virK*	37	17
pgdA::tetRA	*virK*	4**	1
**Experiment 2**			
M90T	*virK*	20	2
ΔpgdA	*virK*	9**	1
**Experiment 3**			
M90T	*virK*	16	3
pgdA::kan	*virK*	17	6

Figure S2: Polarity of *pgdA* mutants. RT-qPCR was performed to establish the effect of three *pgdA* mutations on transcription of the downstream gene *virK*. Three replicates were used to establish the mean number of transcripts per 1000 16S rRNA transcripts and a standard deviation. Strains that were analyzed together are separated by horizontal lines. Probability of being the same as M90T-Sm: * = 0.05 > P > 0.01, ** = 0.01 > P > 0.001 (see Materials and Methods for statistical methods).
