# Supplementary material for: A Shigella flexneri Virulence Plasmid Encoded Factor Controls Production of Outer Membrane Vesicles
Source: G3 (Bethesda). 2014 Nov 5;4(12):2493–503. doi: 10.1534/g3.114.014381 (PMC4267944; doi:10.1534/g3.114.014381)
Supplement: Supporting Information [file supp_g3.114.014381_FigureS3.ps]

## S3

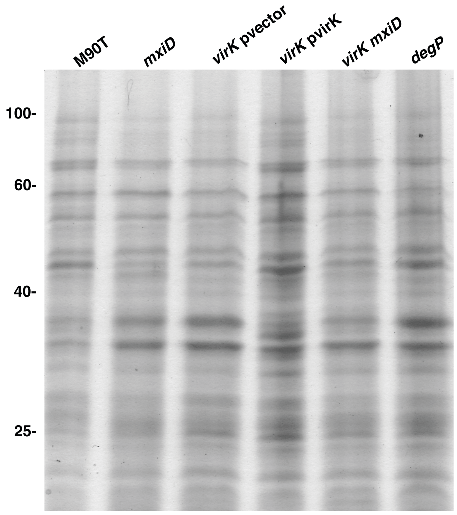


**Figure S3: Loading control of total protein extracts taken from cultures used for protein secretion profiles (Figure 2).** Coomassie-stained SDS-PAGE gels on which crude extracts from bacteria used to collect secreted proteins have been run serve as loading controls.
