# Supplementary material for: A Shigella flexneri Virulence Plasmid Encoded Factor Controls Production of Outer Membrane Vesicles
Source: G3 (Bethesda). 2014 Nov 5;4(12):2493–503. doi: 10.1534/g3.114.014381 (PMC4267944; doi:10.1534/g3.114.014381)
Supplement: Supporting Information [file supp_g3.114.014381_FigureS4.ps]

S4

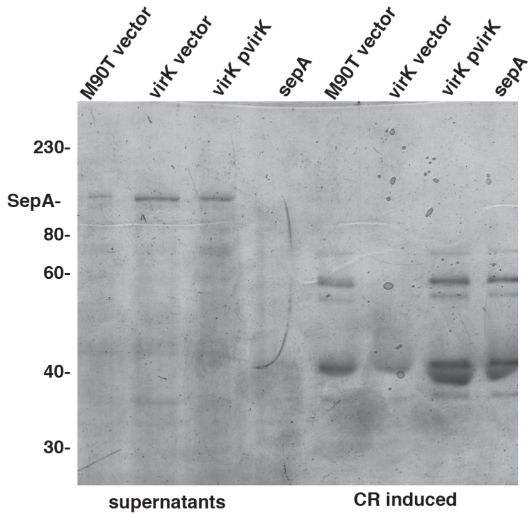

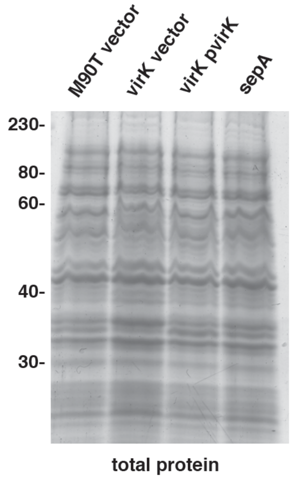

Figure S4: Identification of SepA as major secreted species. Coomassie stained SDS-PAGE gels show the profiles of secreted proteins from wild-type *S. flexneri* bearing an empty vector control (pBluescript) along with the mutants *virK* bearing an empty vector control (pBluescript) and *virK* bearing pvirK, and a *sepA::tet* mutant. The lower image is a Coomassie-stained SDS-PAGE gels on which crude extracts from bacteria used to collect secreted proteins have been run serve as loading controls.
