# Supplementary material for: A Shigella flexneri Virulence Plasmid Encoded Factor Controls Production of Outer Membrane Vesicles
Source: G3 (Bethesda). 2014 Nov 5;4(12):2493–503. doi: 10.1534/g3.114.014381 (PMC4267944; doi:10.1534/g3.114.014381)
Supplement: Supporting Information [file supp_g3.114.014381_TableS1.ps]

Table S1

Primer	Sequence
Tet 2F-PstI	GTACTTCTGCAGCTAAGCACTTGTCTCCTG
Tet 2R-PstI	AGTCTTCTGCAGTTAAGACCCACTTTCACATT
T3 LR1R HindIII	ATGCTAAAGCTTTGTAGGCTGGAGCTGCTTCGAAGTTCCTA
T7 LR1R KpnI	TCGTTAGGTACCATTCCGGGGATCCGTCGACCGAAGTTCCT
P1	ATTCCGGGGATCCGTCGACC
P2	TGTAGGCTGGAGCTGCTTCG
ospG RT1F	TAATAGGTCAGGGGTCGACA
ospG RT1R	CTTGCCAAGCCATTTCCAGA
ipaH4.5 RT1F	GACTGGGCTAGGGAAGGAAC
ipaH4.5 RT1R	GCAAAGGGAGTGGTGGTAAAG
ipgE RT1F	GCCCTTTTTGTGCATTGCCTG
ipgE RT1R	ATTTCCGCCTTCATCATCAG
mxiG RT1F	CACAAAATGATGCGGTATGG
mxiG RT1R	TAAATGGGAATCGTTGATTG
ipaH9.8-Ntermdelfor	GTAATTTCCTCACTGAGCTACCAGCATTTTCTGAGGGAAATA
ipaH9.8-C337delrev	ACCAGGAGGGTTTTCCGGAGATTGTTCCATGTGAGCGCGAC
virK 1F-XbaI	GAATTCTCTAGATATGTTTTCTGTAAGTAACTTATC
virK 1R-SalI	CTCGAGGTCGACTTAATTTAAGTCCTGATGTTCT
