# Supplementary material for: A Shigella flexneri Virulence Plasmid Encoded Factor Controls Production of Outer Membrane Vesicles
Source: G3 (Bethesda). 2014 Nov 5;4(12):2493–503. doi: 10.1534/g3.114.014381 (PMC4267944; doi:10.1534/g3.114.014381)
Supplement: Supporting Information [file supp_g3.114.014381_TableS2.ps]

# Table S2

| Plasmid Name | Description | Forward Primer | Reverse Primer |
|---|---|---|---|
| pRR003 | Encodes Flippase machinery used to catalyze recombination between FRT sites and remove *tetRA* . | N/A[B] | N/A[B] |
| pRR007 | Encodes λ-red mediated recombination machinery and kanamycin resistance. | N/A[B] | N/A[B] |
| pRR008 | Encodes λ-red mediated recombination machinery and gentamycin resistance. | N/A[B] | N/A[B] |
| tetRA-pGEM | *tetRA* fragment from TH2788 (Karlinsey, 2007) ligated into pGEM-T Easy (Promega) | T3 LR1R HindIII[B] | T7 LR1R KpnIB |
| pvirK | *virK* coding sequence cloned into pBluescript II SK(+) | *virK* 1F-XbaI | *virK* 1R-SalI |

[A]See Table S1 for primer sequences
[B]See Material & Methods for plasmid construction details.
